# Hypertension-diabetes comorbidity in tropical Chinese adults

**DOI:** 10.7189/jogh.16.04091

**Published:** 2026-03-06

**Authors:** Jinting Zou, Junliang Tao, Juan Jiang, Changfu Xiong, Bin He, Dingwei Sun, Ying Liu, Dongxian Zhang

**Affiliations:** 1 Hainan Medical University, School of Public Health, Haikou, China; 2 Hainan Medical University, School of Basic Medical Sciences, Haikou, China; 3 Hainan Provincial Center for Disease Control and Prevention, Institute of Tropical Diseases and Chronic Disease Control, Haikou, China

## Abstract

**Background:**

Hypertension-diabetes comorbidity (HDC) substantially increases the risk of cardiovascular and microvascular complications, yet population-based evidence from tropical island settings in China remains limited. This study estimated the prevalence of HDC among adults in Hainan Province and examined associated factors across sociodemographic, metabolic, and lifestyle domains.

**Methods:**

We analysed data from the Hainan Province ‘2 + 3’ health service package epidemiological survey, a population-based cross-sectional study conducted across all 24 administrative divisions from 3 November to 31 December 2022 using a two-stage disproportionate cluster sampling design. After prespecified data quality control and cleaning, 32 857 adults were included. Survey weights were applied to produce population-representative estimates, and survey-weighted multivariable logistic regression was used to identify factors associated with HDC.

**Results:**

The weighted prevalence of HDC was 7.0%, higher in men (8.0%) than women (6.0%), and increased sharply with age (1.6% in 18–39 years, 7.7% in 40–59 years, and 18.5% in ≥ 60 years). In the fully adjusted model, female sex was inversely associated with HDC (adjusted odds ratio (aOR) = 0.64; 95% confidence interval (CI) = 0.55–0.74), while older age (40–59 years: aOR = 5.42; 95% CI = 4.29–6.85), (≥ 60 years: aOR = 16.37; 95% CI = 13.38–20.04) and Li ethnicity (aOR = 1.60; 95% CI = 1.20–2.14) were associated with higher odds. Overweight (aOR = 1.83; 95% CI = 1.63–2.06), obesity (aOR = 2.68; 95% CI = 2.28–3.15), and dyslipidaemia (aOR = 1.74; 95% CI = 1.58–1.92) were independently associated with HDC, whereas underweight showed an inverse association (aOR = 0.67; 95% CI = 0.52–0.86). Among participants with self-reported diagnosed hypertension and diabetes, 66.8% reported taking any blood pressure control measure and 36.9% reported taking any blood glucose control measure, respectively.

**Conclusions:**

In tropical China, HDC affects a substantial proportion of adults and is strongly associated with male sex, older age, excess adiposity, and dyslipidaemia. The low uptake of diabetes control measures among diagnosed individuals highlights the need to strengthen integrated screening, follow-up, and chronic disease management in this setting.

Globally, chronic non-communicable diseases (NCDs) are imposing an escalating disease burden on public health, driven by rapid lifestyles changes and population aging. Hypertension and diabetes, in particular, have reached alarmingly high prevalence levels. Relevant studies indicated that 1.38 billion adults, representing 31.1% of the global adult population, were affected by hypertension [1]. Between 2007 and 2017, the estimated loss of life years attributable to ischemic heart disease and stroke surged by 17.3% and 12%, respectively [2,3]. In 2021 alone, diabetes contributed to 6.7 million deaths worldwide, while diabetes-related health care expenditures skyrocketed from 232 billion USD in 2007 to 966 billion USD in 2021, and the projections suggested that the global diabetic population would rise to 643 million by 2030, with health care costs exceeding 1.03 trillion USD [4]. Of particular concern is China’s worsening diabetes epidemic. According to the Global Burden of Disease (GBD 2021) database, the diabetes mortality rate in China has more than doubled, rising from 6.14 per 100 000 population in 1990 to 12.54 per 100 000 in 2021 [5]. These dramatic increase underscores the urgent need for effective interventions to mitigate the growing NCD burden.

Hypertension and diabetes are two closely interrelated metabolic disorders, with hypertension-diabetes comorbidity (HDC) referring to their concurrent presence in patients. Diabetes arises from impaired insulin secretion or action, resulting in hyperglycaemia due to dysregulated blood glucose control [6]. On the other hand, hypertension is recognised as the leading modifiable risk factor for cardiovascular diseases worldwide [7]. When these conditions coexist, they synergistically elevate the risk of complications including cardiovascular diseases, nephropathy, and retinopathy [8]. This comorbidity not only severely compromises patients' quality of life but also poses a significant challenge to health care systems [9–11]. Multiple epidemiological surveys have shown that over two-thirds of diabetic patients also suffer from hypertension, further amplifying disease burden [12,13]. Alarmingly, their collective contribution to rising global premature mortality rates, with this trajectory showing no signs of abatement [7,14].

Hainan Province, situated in China's southernmost tropical zone, boasts a year-round temperate climate, lush forest ecosystems, and unique geographical characteristics as an island province. Its distinctive island ecosystem, coupled with specific lifestyle patterns, dietary customs, and demographic features, establishes Hainan as a critical research hub for investigating HDC [15]. Surveys indicated that the disease spectrum in Hainan Province is dominated by NCDs, with hypertension and diabetes ranking as the two most prevalent conditions [16]. Notably Hainan's dual status as a premier international tourist destination and a wintering haven for seasonal elderly migrants creates unique population dynamics. Annually, owing to its warm climate, Hainan Province attracts a large number of seasonal elderly migrants that come to spend the winter. This cyclical population mobility, combined with accelerating population aging, exacerbates the challenges in HDC prevention and management. Despite these pressing needs, scientific research on HDC patterns in Hainan remains conspicuously absent in current medical literature [17].

This study aimed to investigate the prevalence of HDC among adults in Southern China and identify its associated risk factors across three dimensions:

1) demographic characteristics, including sex, age, ethnicity, urban-rural residence, education level, marital status;

2) metabolic-related indicators, including body mass index (BMI) and lipid profiles; and

3) lifestyle behaviours, including smoking status, drinking status, second-hand smoke exposure, and physical activity intensity.

By systematically analysing these multidimensional determinants, this study seeks to establish an evidence-based foundation for optimising HDC prevention and control strategies in Hainan Province, while providing translatable insights for public health interventions in other regions with comparable epidemiological profiles.

## METHODS

### Study population and design

This study was based on data from the Hainan Province ‘2 + 3′ health service package epidemiological survey, covering hypertension, diabetes, tuberculosis, hepatitis, and severe mental disorders. The parent survey was not designed specifically for HDC considerations of feasibility for conducting epidemiological surveys for multiple diseases at the same time. While balancing scientific rigor and feasibility, and to save the costs of mobilisation and publicity for field investigations, the ‘2 + 3’ epidemiological sampling survey simultaneously conducted surveys on hypertension, diabetes, tuberculosis, and viral hepatitis. The 2010 tuberculosis epidemiological survey in Hainan Province with 7721 participants showed that the prevalence of active pulmonary tuberculosis among people aged 15 years and above was 539/100 000; the 2018 epidemiological survey on hypertension and diabetes in Hainan Province with 3690 participants showed that the prevalence of hypertension and diabetes among residents aged 18 years and above was 18.70 and 11.30%, respectively; the 2021 hepatitis infection survey in Hainan Province with 1931 participants showed that the HBsAg positivity rate of hepatitis B among people aged 1–69 years was 5.64%. Given that the prevalence of tuberculosis is lower than that of hypertension, diabetes, and hepatitis B, the ‘2 + 3’ epidemiological sampling survey used the tuberculosis prevalence base as the basis for sample size calculation for this survey.

Eligible participants were:

1) adults aged ≥ 18 years;

2) permanent residents with Hainan household registration (excluding those absent from the province for ≥ 6 months); or

3) non-local residents living continuously in Hainan for ≥ 6 months.

The minimum sample size was calculated using the Cochran formula:



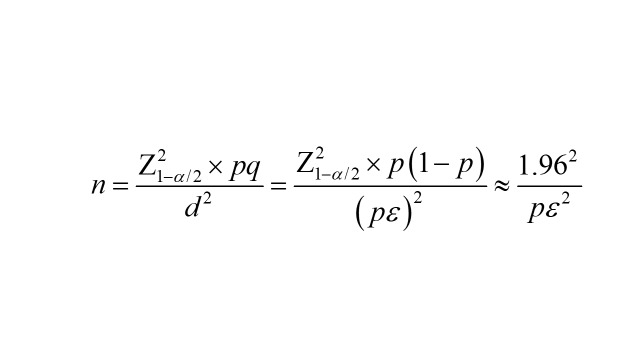



According to previous national tuberculosis epidemiological survey results, data from the three national tuberculosis epidemiological surveys in 1990, 2000, and 2010 showed that the annual decline rate of bacteriologically positive prevalence was 3.2%; based on this, the prevalence in Hainan Province in 1990 was 826/100 000, and the estimated prevalence in 2022 was approximately 404/100 000. Assuming an allowable error of 25%, the sample size calculated by the formula was 15 214. Because this sampling was cluster sampling, the design effect was set to 2, and the non-response rate was 10%. After calculation, the required sample size was 33 809, which was rounded to 33 810; that is, surveying 33 810 people could simultaneously meet the representativeness requirements for hypertension, diabetes, tuberculosis, and viral hepatitis. To improve precision and feasibility, 30 survey sites were selected, with a minimum of 1127 participants per site.

A population-based cross-sectional survey was conducted across all 24 administrative divisions in Hainan Province from 3 November to 31 December 2022, using a two-stage disproportionate cluster sampling design. In the first stage, at least one survey site was allocated to each division, and the remaining sites were allocated proportionally to population size; six high-population divisions (Danzhou City, Longhua District, Meilan District, Qiongshan District, Wanning City, and Wenchang City) were allocated two sites each, and the remaining 18 divisions were allocated one site each (total 30 sites). In the second stage, village/neighbourhood committees were numbered and randomly selected within each division according to the allocated number of sites, with a minimum threshold of 1127 participants per selected committee. Sampling was conducted using SAS for Windows 9.4 after compiling the provincial population database.

During sampling, one selected site was replaced after expert consultation; additionally, five selected committees had resident populations below 1127 and could not be substituted, and therefore five supplementary committees were added. Thus, 35 committees (30 initial + 5 supplementary) were surveyed. After a prespecified data quality control and cleaning process, excluding records with incomplete key variables or implausible values, 32 857 participants were included in the final analytic sample. All statistical analyses in this study were adjusted using complex survey weighting to enhance the provincial representativeness of the findings. The weighting system comprised three components: base design weights calculated using SAS version 9.4 based on the sampling probabilities at each stage to reflect the randomness of the sampling design; non-response adjustment weights constructed based on the proportion of actual participants to mitigate bias from non-sampling errors; and post-stratification weights based on the 2020 Seventh National Population Census data of Hainan Province, which standardised the sex and age structure to ensure high consistency with the standard provincial population. To enhance statistical efficiency and prevent interference from extreme weight values, the final weights were truncated at 0.5 and 2.0. All prevalence estimates and multivariable analysis results reported in this paper are weighted adjusted data.

The study protocol was approved by the Ethics Review Committee of Hainan Provincial Center for Disease Control and Prevention (approval number: 2024041).

### Data collection

#### Questionnaire survey

Questionnaire data were collected through a combination of household appointments and centralised survey sessions. Sociodemographic information included sex, age group, ethnicity (Han, Li, and other minorities), urban/rural residence, educational attainment, and marital status. Lifestyle information, including smoking status, second-hand smoke exposure, drinking status, and physical activity intensity was self-reported by participants.

Smoking status was classified based on the question ‘Do you currently smoke?’ Participants who answered ‘Yes, every day/Yes, but not every day’ were categorised as current smokers; those who reported having smoked previously but not currently were categorised as former smokers; and those who reported never smoking were categorised as never smokers. Second-hand smoke exposure was stratified according to the reported number of days per week exposed to second-hand smoke as no exposure, low-frequency exposure (1–3 days/week on average), or high-frequency exposure (4–7 days/week on average). Drinking status was determined based on alcohol consumption in the past 12 months and the most recent time of drinking, and categorised as drinking within the past 30 days, past drinking (> 30 days ago), or no drinking. Physical activity intensity was classified according to the questionnaire as no moderate- or vigorous-intensity activity, vigorous-intensity only, moderate-intensity only, or moderate- plus vigorous-intensity activity.

#### Physical examination

Physical examination included height, weight, waist circumference (WC), and blood pressure, which were measured by standardised trained investigators. Height was measured using a TZG Type Height Gauge, weight was measured using a G&G TB-200K Electronic Scale, WC was measured using a nonelastic tape, and blood pressure was measured and recorded using a calibrated electronic sphygmomanometer.

#### Laboratory testing

Laboratory testing included fasting/postprandial blood glucose and lipid profiles. For all participants aged ≥ 18 years, fasting venous blood samples (2 mL) were collected using sodium fluoride/potassium oxalate anticoagulant tubes and analysed for glucose levels via the hexokinase method, while postprandial glucose levels were measured two hours after standardised glucose intake. Lipid profiles, including total cholesterol (TC), triglycerides (TG), low-density lipoprotein cholesterol (LDL-C), and high-density lipoprotein cholesterol (HDL-C), were assessed at designated flow control stations: TC and TG were quantified through enzymatic assays, whereas LDL-C and HDL-C were determined via direct measurement methods.

### Indicator definition

According to the Chinese Guidelines for Hypertension Prevention and Treatment (2018 edition), [18] the diagnostic criteria for adult hypertension are defined as: systolic blood pressure (SBP)≥ 140 mmHg and/or diastolic blood pressure (DBP) ≥ 90 mmHg in individuals not using anti-hypertensive medications. Blood pressure measurements followed a standardised protocol: three consecutive readings were taken at 1-minute intervals, with the average of the final two readings recorded as the definitive value. Hypertensive participants included those meeting the above blood pressure thresholds, or those with a prior physician diagnosis confirmed by township/community-level (or higher) health care facilities.

According to the Chinese Guidelines for the Prevention and Control of Type 2 Diabetes (2017 edition), [19] the diagnostic criteria for adult diabetes are defined as: fasting plasma glucose (FPG) ≥ 7.0 mmol/L and/or 2-hour postprandial glucose (OGTT–2 h) ≥ 11.1 mmol/L, or a prior diagnosis confirmed by health care facilities at or above the township/community level.

According to the World Health Organization (WHO) BMI classification, [20] individuals are categorised as follows: Underweight is defined as BMI < 18.5, normal weight as BMI = 18.5–24.9, overweight as BMI = 25–29.9, and obese as BMI ≥ 30.

According to the Chinese Guidelines for Lipid Management (2023), [21] dyslipidaemia is diagnosed based on the following biochemical thresholds: TC ≥ 6.2 mmol/L was considered as hypercholesterolemia, TG ≥ 2.3 mmol/L was considered as hypertriglyceridemia, HDL-C < 1.0 mmol/L was considered as hypoalphalipoproteinemia, and LDL-C ≥ 4.1 mmol/L was considered as hyperbetalipoproteinemia. Participants were classified as dyslipidaemic if they met ≥ 1 of these criteria or had a prior dyslipidaemia diagnosis confirmed by township/community-level (or higher) health care institutions.

### Data collection procedure

After trained staff explained the survey's purpose, procedures, and methods, participants provided informed consent by signing the Informed Consent Form (ICF); those unable to read or write, or physically unable to sign, provided thumbprints as legally valid alternatives. Staff then documented participants' identity information, collected venous blood samples, and distributed Field Survey Flow Sheets to coordinate questionnaire administration, physical measurements, and blood sample collection. All collected data are filled out and entered into the information system by trained staff to ensure authenticity and accuracy.

### Quality control

#### Preparation stage

Municipal and county-level authorities in Hainan Province developed region-specific implementation plans in alignment with the provincial public health guidelines. Provincial CDC-convened experts conducted standardised competency training for all field staff, which required certification prior to operational deployment. Key preparatory tasks included Validate the target population registry and survey sites to ensure geographic consistency, and implementing a pilot survey in one randomly selected county to optimise on-site survey protocols. This systematic approach guaranteed methodological uniformity across all study sites.

#### Implementation at site stage

The survey site was partitioned into designated functional zones: registration, phlebotomy, anthropometry, questionnaire administration, and postprandial glucose monitoring (sugar consumption area). Participants sequentially progressed through these zones as per their Field Survey Flow Sheets to prevent procedural omissions. During questionnaire administration, staff adhered to neutral probing protocols without suggestive or leading inquiries, while concurrently verifying response completeness and the accuracy of the data entry. All blood specimens were collected, processed, transported, and analysed in strict compliance with laboratory standards.

#### Acceptance stage

The study required a 95% field acceptance threshold, defined as the proportion of completed surveys meeting predefined validity criteria. Subsequently, the data collected is again reviewed and cleaned.

### Statistical analysis

#### Data analysis

Sampling and survey weighting were implemented in SAS for Windows version 9.4 (SAS Institute Inc., Cary, NC, USA) after compiling the provincial population database. The final weights consisted of:

1) base design weights derived from stage-specific sampling probabilities to account for the multistage design,

2) non-response adjustment weights based on the ratio of actual participants to intended samples to reduce non-sampling error, and

3) post-stratification weights calibrated to the sex- and age-specific distribution from the 2020 Seventh National Population Census of Hainan Province to align the sample with the provincial population structure.

Tabular data management and table preparation were performed using WPS Office, Excel tables (Kingsoft Office Software, Zhuhai, China). Descriptive analyses and logistic regression under the complex sampling framework were conducted in IBM SPSS Statistics version 26.0 (IBM Corp., Armonk, NY, USA). Forest plots were generated using *R*, version 4.4.2 (R Foundation for Statistical Computing, Vienna, Austria).

#### Analytical pipeline

This study was based on data from the Hainan Province ‘2 + 3’ health service package epidemiological survey, which covered hypertension, diabetes, tuberculosis, hepatitis, and severe mental disorders. The parent survey was not designed specifically for hypertension-diabetes comorbidity (HDC). The overall target sample size was calculated using tuberculosis as the reference condition, given its comparatively low expected prevalence, to ensure provincial representativeness and adequate precision. Although the sample size determination was based on tuberculosis, the overall sample size of this survey was sufficiently large to support analyses of hypertension and diabetes with adequate statistical precision.

Given the two-stage disproportionate cluster sampling design, all analyses were conducted using complex survey methods with weighting. Survey weights were constructed by deriving base weights as the inverse of the product of selection probabilities across sampling stages, then adjusting for non-response, and finally post-stratifying by age and sex to the 2020 Hainan Seventh National Population Census (with trimming of extreme weights), to obtain population-representative estimates. Descriptive results are presented as weighted percentages with 95% confidence intervals (95% CIs).

Group differences in categorical variables were evaluated using design-based Rao-Scott adj. F tests. Associations between HDC (and related outcomes) and candidate indicators were examined using survey-weighted multivariable logistic regression models, reporting adjusted odds ratios (aORs) and 95% CIs. Covariates were entered sequentially in blocks (sociodemographic, metabolic, and lifestyle factors) to assess the robustness of estimates across Models 1–3(M1–M3). To reduce potential multicollinearity among adiposity-related indicators, BMI categories were used as the primary anthropometric measure in the main models. A two-sided *P*-value < 0.05 was considered statistically significant. All analyses were performed using the Complex Samples module of IBM SPSS Statistics.

Definitions of outcomes, covariate sets, and the unweighted analytic sample size for each prespecified model (M1–M8) are summarised in Table S1 in the **Online Supplementary Document**. The stepwise models (M1–M3) are provided in Table S2–3 in the **Online Supplementary Document**. Sensitivity analyses were conducted to examine the robustness of the main findings by:

1) redefining the outcome using alternative case-control contrasts (HDC *vs*. none; HDC *vs*. only hypertension/diabetes; only hypertension *vs*. none; and only diabetes *vs*. none) and

2) using objectively measured HDC as the outcome definition; results are presented in Table S4–5 in the **Online Supplementary Document**. Multicollinearity was assessed after dummy-coding categorical predictors using tolerance and variance inflation factors (VIFs), and no evidence of problematic multicollinearity was observed (maximum VIF = 3.06; minimum tolerance = 0.33).

## RESULTS

### Descriptions of sample demographic information

Among all 32 857 participants, 15 124 (46.1%) were males and 17 733 (54.1%) were females. A total of 3306 participants had HDC (weighted prevalence 7.0%), while 29 551 participants did not have HDC (weighted 93.0%). The weighted prevalence of HDC was higher in males (8.0%) than in females (6.0%) and increased markedly with age (1.6% in 18–39 years, 7.7% in 40–59 years, and 18.5% in ≥ 60 years). Most participants were Han (75.5%), lived in the countryside (74.2%), had junior high school education (38.7%), and were married/cohabiting (88.6%). Based on the Rao-Scott adjusted F test, the weighted prevalence of HDC differed significantly across sex, age group, education status, marital status, BMI, blood lipid status, smoking status, second-hand smoke exposure, and physical activity intensity (all *P* < 0.05), but not by ethnicity, urban/rural residence, or drinking status. The participants’ general sociodemographic characteristics are summarised in **Table 1**.

**Table 1:** Characteristics of the HDC population

Variables	Unweighted, n (%)	Weighted HDC prevalence, % (95%CI)	Weighted, Non-HDC, % (95% CI)	Rao-Scott adj.F (df1,df2)	*P-*value
**Sex**					
Male	15 124 (46.1)	8.0 (7.1–9.0)	92.0 (91.0–92.9)	25.203 (1,12)	<0.001
Female	17733 (54.1)	6.0 (5.0–7.2)	94.0 (92.8–95.0)		
**Age group (years)**					
18–39	7670 (23.3)	1.6 (1.2–2.1)	98.4 (97.9–98.8)	430.583 (1.515,18.183)	<0.001
40–59	14 597 (44.4)	7.7 (6.6–8.9)	92.3 (91.1–93.4)		
≥60	10 590 (32.2)	18.5 (16.7–20.3)	81.5 (79.7–83.3)		
**Ethnicity**					
Han	24 805 (75.5)	7.1 (6.0–8.3)	92.9 (91.7–94.0)	0.159 (1.155,13.862)	0.732
Li	7470 (227)	6.9 (5.2–9.2)	93.1 (90.8–94.8)		
Other	582 (1.8)	8.5 (6.4–11.1)	91.5 (88.9–93.6)		
**Urban/rural**					
Town	8474 (25.8)	7.6 (5.7–10.1)	92.4 (88.9–94.3)	0.361 (1,12)	0.559
Countryside	24 383 (74.2)	6.8 (5.7–8.2)	93.2 (91.8–94.3)		
**Education status**					
Illiterate	3929 (12.0)	12.0 (8.7–16.3)	88.0 (83.7–93.1)	24.730 (2.081,24.970)	<0.001
Primary school	8059 (24.5)	9.8 (9.1–10.6)	90.2 (89.4–90.9)		
Junior high school	12 727 (38.7)	6.0 (5.2–6.9)	94.0 (93.1–94.8)		
High school	5562 (16.9)	6.2 (4.9–7.8)	93.8 (92.2–95.1)		
University or above	2580 (7.9)	3.5 (2.5–4.9)	96.5 (95.1–97.5)		
**Marital status**					
Unmarried	2606 (7.9)	2.9 (1.8–4.5)	97.1 (95.5–98.2)	35.510 (1.040,12.485)	<0.001
Married/cohabitation	29 117 (88.6)	7.7 (6.7–8.7)	92.3 (91.3–93.3)		
Divorce/widowhood	1134 (3.5)	17.0 (14.9–19.2)	83.0 (80.8–85.1)		
**BMI (kg/m^2^)**					
Normal	19 993 (60.8)	5.8 (5.1–6.8)	94.2 (93.2–94.9)	83.327 (2.555,30.660)	<0.001
Underweight	1998 (6.1)	3.2 (2.5–4.1)	96.8 (95.9–97.5)		
Overweight	9273 (28.2)	10.1 (8.6–11.9)	89.9 (88.1–91.4)		
Obese	1590 (4.8)	11.0 (9.2–13.0)	89.0 (87.0–90.8)		
**Blood lipids**					
Normal	18 890 (57.5)	4.9 (4.3–5.6)	95.1 (94.4–95.7)	681.863 (1,12)	<0.001
Abnormal	13 922 (42.4)	10.4 (9.2–11.8)	89.6 (88.2–90.8)		
**Smoking**					
Never smoked	22 340 (68.0)	6.9 (5.8–8.1)	93.1 (91.9–94.2)	31.796 (1.176,14.108)	<0.001
Current smoker	8997 (27.4)	6.6 (5.9–7.5)	93.4 (92.5–94.1)		
Former smoker	1520 (4.6)	14.0 (12.4–15.8)	86.0 (84.2–87.6)		
**Second-hand smoke exposure**					
None	11 192 (34.1)	8.9 (7.4–10.6)	91.1 (89.4–92.6)	24.742 (1.881,22.571)	<0.001
Low frequency	4084 (12.4)	5.5(4.6–6.6)	94.5 (93.4–95.4)		
High frequency	16 119(49.1)	6.4 (5.8–7.2)	93.6 (92.8–94.2)		
**Drinking**					
No drinking in the past 12 mo	19 270 (58.6)	7.1 (6.0–8.4)	92.9 (91.6–94.0)	1.143 (1.388,16.654)	0.322
Former drinking (no drinking in the past 30 d)	3210 (9.8)	6.2 (5.2–7.2)	93.8 (92.5–94.8)		
Drinking in the past 30 d	10 377 (31.6)	7.2 (6.2–8.0)	92.8 (91.6–94.0)		
**Physical activity intensity**					
No vigorous, or moderate-intensity activity	8608 (26.2)	8.5 (6.8–10.7)	91.8 (89.3–92.7)	5.737 (1.311,15.735)	0.022
Vigorous-intensity activity only	1052 (3.2)	7.3 (6.0–8.7)	92.7 (91.3–94.0)		
Moderate-intensity activity only	13 300 (40.5)	6.6 (5.5–7.8)	93.4 (92.2–94.5)		
Both vigorous- and moderate-intensity activity	9897 (30.1)	6.3 (5.6–7.1)	93.7 (92.9–94.4)		

CI – confidence interval, F – F statistic, HDC – hypertension–diabetes comorbidity

### Logistic regression analyses of factors associated with HDC

**Figure 1:**
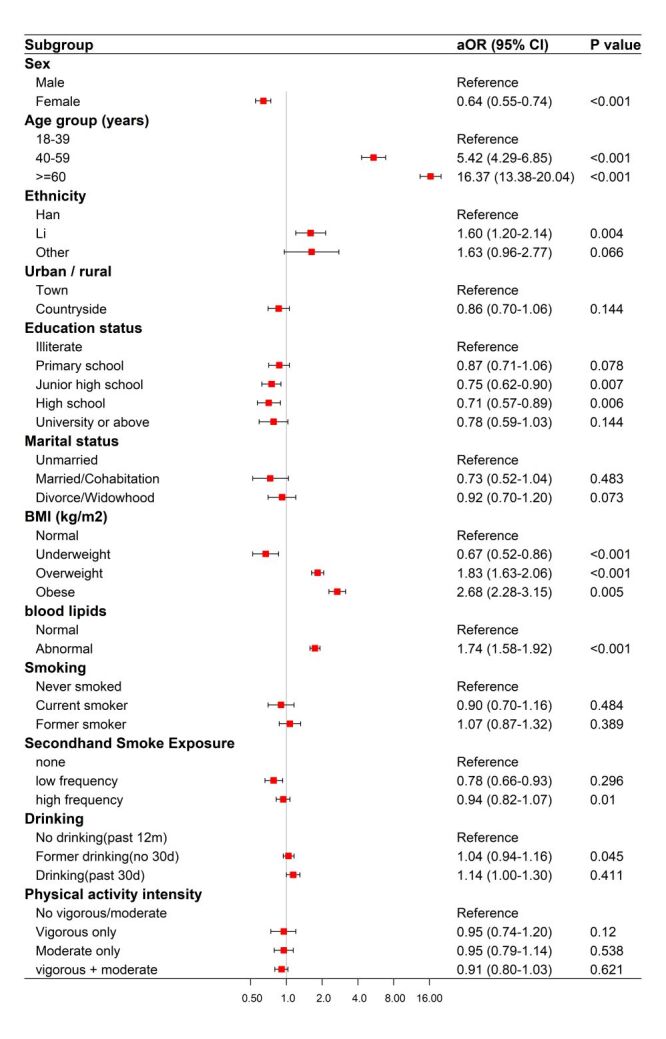
Adjusted odds ratios for hypertension–diabetes comorbidit (HDC) from logistic regression (M3).

Based on the fully adjusted survey-weighted multivariable logistic regression model (M3), female sex was inversely associated with HDC compared with male sex (aOR = 0.64; 95% CI = 0.55–0.74). Relative to participants aged 18–39 years, those aged 40–59 years (aOR = 5.42; 95% CI = 4.29–6.85) and ≥60 years (aOR = 16.37; 95% CI = 13.38–20.04) had higher odds of HDC. With respect to ethnicity, Li participants had higher odds of HDC than Han participants (aOR = 1.60; 95% CI = 1.20–2.14), whereas the estimate for other ethnicities was less precise (aOR = 1.63; 95% CI = 0.96–2.77). Compared with illiterate participants, those with junior high school (aOR = 0.75; 95% CI = 0.62–0.90) and high school (aOR = 0.71; 95% CI = 0.57–0.89) education had lower odds of HDC. Regarding metabolic indicators, underweight was inversely associated with HDC (aOR = 0.67; 95% CI = 0.52–0.86), overweight (aOR = 1.83; 95% CI = 1.63–2.06) and obesity (aOR = 2.68; 95% CI = 2.28–3.15) were associated with higher odds, and abnormal blood lipids were also associated with higher odds of HDC (aOR = 1.74; 95% CI = 1.58–1.92). Estimates were broadly consistent across the stepwise models (M1–M3); therefore, we primarily present the fully adjusted model 3 (**Figure 1**, **Table 2**).

**Table 2:** Associations of demographic, metabolic, and lifestyle factors with HDC (M3)

Variable	Category	aOR (95% CI)	*P*-value
Sex (Ref: male)	Female	0.64 (0.55–0.74)	<0.001
Age group (Ref: 18–39)	40–59	5.42 (4.29–6.85)	<0.001
	≥ 60	16.37 (13.38–20.04)	<0.001
Ethnicity (Ref: Han)	Li	1.6 (1.2–2.14)	0.004
	Other	1.63 (0.96–2.77)	0.066
Urban/rural (Ref: town)	Countryside	0.86 (0.7–1.06)	0.144
Education status (Ref: illiterate)	Primary school	0.87 (0.71–1.06)	0.078
	Junior high school	0.75 (0.62–0.90)	0.007
	High school	0.71 (0.57–0.89)	0.006
	University or above	0.78 (0.59–1.03)	0.144
Marital status (Ref: unmarried)	Married/cohabitation	0.73 (0.52–1.04)	0.483
	Divorce/widowhood	0.92 (0.70–1.20)	0.073
BMI (kg/m^2^) (Ref: normal)	Underweight	0.67 (0.52–0.86)	<0.001
	Overweight	1.83 (1.63–2.06)	<0.001
	Obese	2.68 (2.28–3.15)	0.005
blood lipids (Ref: normal)	Abnormal	1.74 (1.58–1.92)	<0.001
Smoking (Ref: never smoked)	Current smoker	0.90 (0.70–1.16)	0.484
	Former smoker	1.07 (0.87–1.32)	0.389
Secondhand smoke exposure (Ref: none)	Low frequency	0.78 (0.66–0.93)	0.01
	High frequency	0.94 (0.82–1.07)	0.296
Drinking (Ref: No drinking in the past 12 mo)	Former drinking (no drinking in the past 30 d)	1.04 (0.94–1.16)	0.045
	Drinking in the past 30 d	1.14 (1.00–1.30)	0.411
Physical activity intensity (Ref: no vigorous- or moderate-intensity activity)	Vigorous-intensity activity only	0.95 (0.74–1.20)	0.12
	Moderate-intensity activity only	0.95 (0.79–1.14)	0.538
	Both vigorous- and moderate-intensity activity	0.91 (0.80–1.03)	0.621

M3 – Model 3, aOR – adjusted odds ratio, CI – confidence interval, Ref – reference

The effect estimates were broadly consistent in sensitivity analyses using alternative outcome specifications and objectively measured HDC, supporting the robustness of the main findings (Table S4–5 in the **Online Supplementary Document**).

### Management measures among participants with self-reported hypertension and diabetes

**Figure 2:**
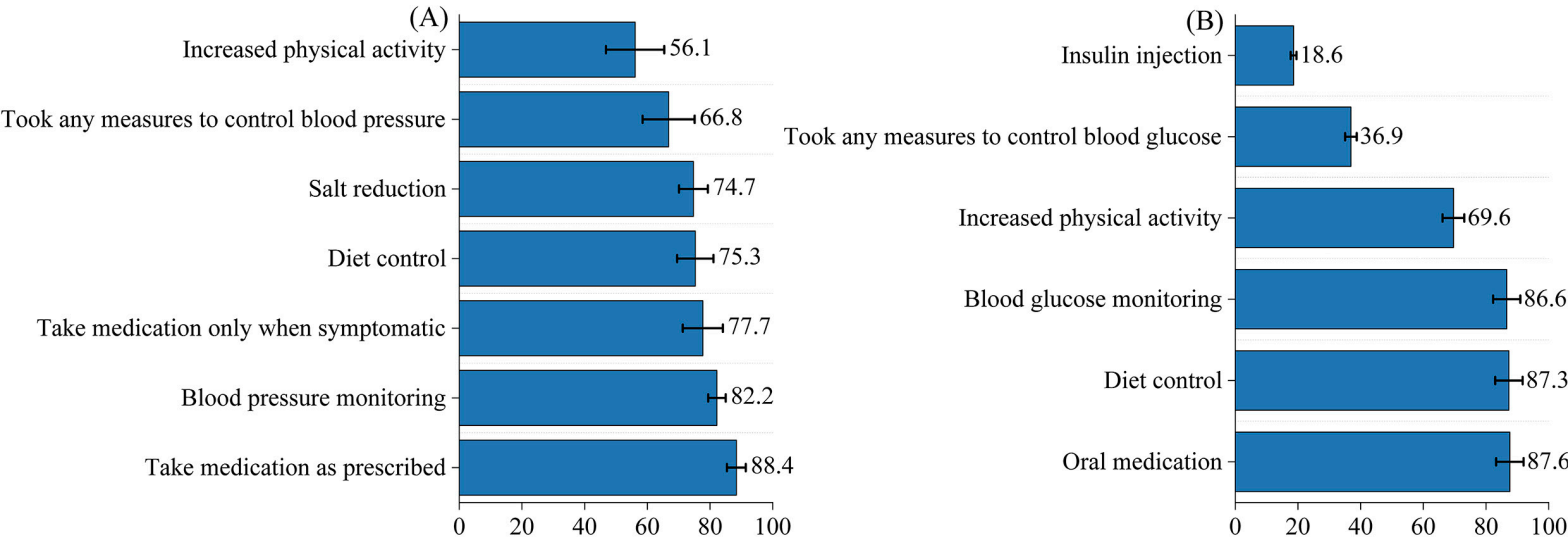
Prevalence of hypertension and diabetes management measures. **Panel A.** Hypertension management measures. **Panel B.** Diabetes management measures.

Among participants with self-reported diagnosed hypertension, 66.8% reported taking any blood pressure control measure (unweighted n = 3619). Among those who reported taking any measures (multiple response), the most commonly reported actions were taking medication as prescribed (88.4%, 95% CI = 85.4–90.9), blood pressure monitoring (82.2%, 95% CI = 79.4–84.7), and taking medication only when symptomatic (77.7%, 95% CI = 71.3–83.0), followed by diet control (75.3%, 95% CI = 69.5–80.4) and salt reduction (74.7%, 95% CI = 70.1–78.8). Increased physical activity was reported less frequently (56.1%, 95% CI = 46.8–64.9) (**Figure 2**, Panel A, **Table 3**).

**Table 3:** Disease management among self-reported diagnosed hypertension/diabetes

Indicator	Category	Unweighted (n)*	Weighted (%)*	95% CI
**Hypertension management among self-reported physician-diagnosed hypertension (n = 5057)†**				
Took any measures to control blood pressure	Yes	3619	66.8	58.5–74.1
BP control measures (multiple response): take medication as prescribed	Yes	3222	88.4	85.4–90.9
BP control measures (multiple response): take medication only when symptomatic	Yes	2843	77.7	71.3–83.0
BP control measures (multiple response): diet control	Yes	2716	75.3	69.5–80.4
BP control measures (multiple response): salt reduction	Yes	2684	74.7	70.1–78.8
BP control measures (multiple response): increased physical activity	Yes	2073	56.1	46.8–64.9
BP control measures (multiple response): blood pressure monitoring	Yes	2988	82.2	79.4–84.7
**Diabetes management among self-reported physician-diagnosed diabetes (n = 2365)‡**				
Took any measures to control blood glucose	Yes	835	36.9	30.60–43.80
Glucose control measures (multiple response): oral medication	Yes	729	87.6	84.10–90.30
Glucose control measures (multiple response): insulin injection	Yes	158	18.6	12.00–27.50
Glucose control measures (multiple response): diet control	Yes	723	87.3	83.10–90.70
Glucose control measures (multiple response): increased physical activity	Yes	589	69.6	58.20–79.00
Glucose control measures (multiple response): blood glucose monitoring	Yes	727	86.6	82.60–89.80

BP– blood pressure, CI– confidence interval

*n is unweighted and percentages are survey-weighted

†Hypertension.

‡Diabetes.

Among participants with self-reported diagnosed diabetes, 36.9% reported taking any blood glucose control measure (unweighted, n = 835). Among those reporting any measures (multiple response), oral medication (87.6%, 95% CI = 84.1–90.3), diet control (87.3%, 95% CI = 83.1–90.7), and blood glucose monitoring (86.6%, 95% CI = 82.6–89.8) were the most frequently reported, followed by increased physical activity (69.6%, 95% CI = 58.2–79.0); insulin injection was reported by 18.6% (95% CI = 12.0–27.5) (**Figure 2**, Panel B, **Table 3**).

### Factors associated with management measures among participants with self-reported hypertension and diabetes

**Figure 3:**
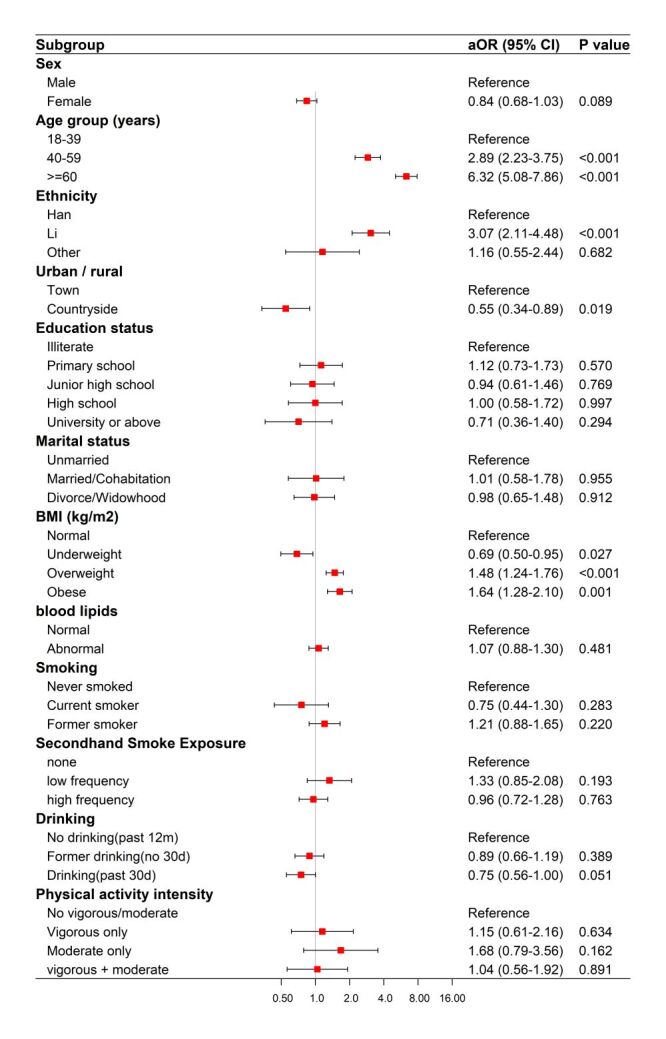
Factors associated with adopting any blood pressure control measure among participants with self-reported hypertension.

Among participants with self-reported physician-diagnosed hypertension, the survey-weighted fully adjusted multivariable logistic regression showed that older age was strongly associated with higher odds of reporting any blood pressure control measure: compared with those aged 18–39 years, participants aged 40–59 years had higher odds (aOR = 2.89; 95% CI = 2.23–3.75, *P* < 0.001) and those aged ≥60 years had even higher odds (aOR = 6.32; 95% CI = 5.08–7.86, *P* < 0.001). Compared with Han ethnicity, Li ethnicity was associated with higher odds (aOR = 3.07; 95% CI = 2.11–4.48, *P* < 0.001). In addition, compared with normal BMI, overweight (aOR = 1.48; 95% CI = 1.24–1.76, *P* < 0.001) and obesity (aOR = 1.64; 95% CI = 1.28–2.10, *P* = 0.001) were associated with higher odds of adopting control measures, whereas underweight was inversely associated (aOR = 0.69; 95% CI = 0.50–0.95, *P* = 0.027). Participants living in the countryside had lower odds than those living in towns (aOR = 0.55; 95% CI = 0.34–0.89, *P* = 0.019). Other covariates, including education, marital status, blood lipids, smoking, second-hand smoke exposure, drinking, and physical activity intensity, were not statistically significantly associated after full adjustment (**Figure 3**; Table S6 in the **Online Supplementary Document**).

Among participants with self-reported physician-diagnosed diabetes, the survey-weighted fully adjusted model indicated that Li ethnicity was associated with lower odds of reporting any blood glucose control measure compared with Han ethnicity (aOR = 0.19; 95% CI = 0.07–0.73, *P* = 0.020). Drinking in the past 30 days was also inversely associated (aOR = 0.66; 95% CI = 0.07–0.93, *P* = 0.022). In contrast, compared with no vigorous/moderate activity, moderate-intensity activity only was associated with higher odds of adopting control measures (aOR = 2.13; 95% CI = 1.48–2.27, *P* = 0.043). No statistically significant associations were observed for sex, age group, residence, education, marital status, BMI category, blood lipids, smoking, second-hand smoke exposure, or other physical activity categories after adjustment (Figure S1 and Table S7 in the **Online Supplementary Document**).

## DISCUSSION

### Main findings of the study

This study yielded the following key findings. First, HDC was more common in men than in women, indicating a higher HDC burden among males. Second, the prevalence of HDC increased markedly with age, with older adults representing the highest-risk group. Third, metabolic factors were strongly associated with HDC: overweight and obesity, as well as dyslipidaemia, were independently associated with higher odds of HDC, whereas underweight was inversely associated. Fourth, compared with illiterate participants, junior high school and high school education were associated with lower odds of HDC, while the association for university education was not statistically significant. Fifth, among participants with self-reported diagnosed hypertension or diabetes, disease management remained suboptimal: only about two-thirds of those with diagnosed hypertension reported taking any blood pressure control measures, and fewer than two-fifths of those with diagnosed diabetes reported taking any blood glucose control measures, highlighting the need to strengthen follow-up and integrated management for diagnosed patients.

### What is already known on this topic?

Hypertension and diabetes frequently co-occur because they share common upstream determinants, including population ageing, excess adiposity, insulin resistance, and dyslipidaemia [22]. Compared with having either condition alone, individuals with both conditions face a substantially higher risk of cardiovascular and renal complications, making early identification and integrated management a public health priority [23].

Population-based evidence from low- and middle-income countries suggests that HDC is already a considerable burden. The STEPS survey in India reported an adult HDC prevalence of about 4.5%, with higher risk among older adults and strong associations with obesity and dyslipidaemia, highlighting the importance of lifestyle-oriented prevention [24]. Similarly, a nationally representative analysis from Bangladesh reported an HDC prevalence of around 4.5%, and consistently showed that overweight and obesity were key correlates of diabetes, hypertension, and their comorbidity [25].

In China, although diabetes and hypertension are highly prevalent, population-based research focusing specifically on HDC remains comparatively limited and shows clear regional heterogeneity. A study from central China reported an HDC prevalence of 2.8% and found that higher BMI and waist-to-height ratio were strongly associated with increased HDC risk [26]. Evidence from southern China, particularly tropical island settings such as Hainan Province, has been scarce, underscoring the need for locally representative estimates and context-specific prevention priorities.

### What this study adds?

Using a large provincially representative sample with complex survey weighting, this study provides new evidence on the burden and correlates of HDC in Hainan Province. We found a weighted HDC prevalence of 7.0%, which is higher than estimates reported in India and Bangladesh (about 4.5%) and also higher than that reported in central China (2.8%) [24–26]. The markedly higher prevalence highlights the need to strengthen prevention and early detection strategies tailored to high-risk groups in this setting.

In the fully adjusted model, we confirmed several independent factors associated with HDC. Male sex and older age were strongly associated with higher odds of HDC. Importantly, the burden of HDC increased steeply with age: the survey-weighted prevalence rose from 1.6% in adults aged 18–39 years to 7.7% in those aged 40–59 years and 18.5% in those aged ≥ 60 years, corresponding to absolute differences of 6.1 and 16.9 percentage points compared with the youngest group. In this context, large age-related odds ratios should be interpreted with caution. When an outcome is not rare – particularly in older age groups – odds ratios from cross-sectional logistic models can diverge substantially from prevalence ratios (and relative risks), inflating the apparent strength of association. Moreover, age in cross-sectional data partly captures longer time-at-risk and the cumulative probability of diagnosis and detection across the life course rather than direct etiologic effects. Therefore, we present age-stratified survey-weighted prevalence and absolute differences alongside odds ratios to facilitate interpretation, and the age-related associations should be viewed primarily as cross-sectional correlates reflecting a pronounced burden gradient.

We confirmed several independent factors associated with HDC. Male sex and older age were strongly associated with higher odds of HDC. Metabolic factors showed robust associations: overweight and obesity, as well as dyslipidaemia, were independently associated with higher odds of HDC, whereas underweight was inversely associated. We also observed an educational gradient, with junior high school and high school education associated with lower odds of HDC compared with illiteracy, while the estimate for university education was not statistically significant. These findings suggest that prevention efforts should prioritise individuals with adverse metabolic profiles and those with low educational attainment, and that health education and risk communication should be designed to be accessible and actionable for people with limited literacy.

Notably, although physical activity is biologically and epidemiologically linked to lower cardiometabolic risk, its association with HDC may be partly mediated through adiposity and metabolic status and can be sensitive to measurement and reverse causation in cross-sectional analyses [27,28]. Extensive longitudinal evidence supports an overall inverse association between physical activity and incident hypertension and type 2 diabetes, although some cohorts have suggested potential nonlinear patterns across activity levels [29].

Beyond estimating prevalence and determinants, this study adds evidence on disease management gaps among individuals with known diagnoses. Among participants with diagnosed hypertension, only 66.8% reported taking any measures to control blood pressure, and among those with diagnosed diabetes, only 36.9% reported taking any measures to control blood glucose. This suboptimal self-management aligns with national evidence showing that awareness, treatment, and control of hypertension and diabetes remain insufficient in China, reinforcing the need to strengthen follow-up, patient education, and integrated management pathways in primary care, especially for older adults, men, and those with obesity or dyslipidaemia [30–33].

### Limitations of this study

This study has several limitations. First, because of the cross-sectional design, causal inference is not possible; thus, temporal ordering cannot be established and causal drivers of hypertension-diabetes comorbidity (HDC) cannot be identified. In addition, because HDC is not rare in older age groups, odds ratios from cross-sectional logistic models may overstate prevalence ratios (and relative risks); therefore, the magnitude of age-related associations should be interpreted cautiously and complemented by absolute differences in survey-weighted prevalence. Second, lifestyle behaviours (*e.g.* smoking, alcohol consumption, second-hand smoke exposure, and physical activity) were assessed using brief self-reported questions, which may be subject to recall bias and social desirability bias. Moreover, because we did not quantify intensity, frequency, duration, or cumulative exposure and instead modelled these behaviours using broad categories, non-differential misclassification is possible, which could bias true associations toward the null and partly explain the null or weak associations observed for these behaviours. Third, the parent ‘2 + 3’ survey was not specifically designed for HDC; therefore, some clinically important information was not collected, and residual confounding may persist. In addition, among participants with self-reported diagnosed hypertension or diabetes, disease management was assessed using self-reported control measures, which may not fully capture actual treatment intensity or objectively measured control status. Fourth, although we employed a province-representative complex sampling design with weighting, the findings may not be fully generalisable beyond Hainan Province.

## CONCLUSIONS

From a public health and policy perspective, our findings support more targeted and operational recommendations. Given the pronounced age gradient and sex differences observed in this study, screening and follow-up for hypertension and diabetes and their comorbidity could be prioritised for older adults and men, with clear referral and follow-up pathways in primary care. In addition, because overweight and obesity and dyslipidaemia were robustly associated with HDC, incorporating obesity indicators such as body mass index categories together with lipid status into routine primary-care risk stratification may help identify populations with a higher HDC burden earlier and enable more proactive prevention and management. Moreover, our results indicate important gaps in disease self-management among individuals with known diagnoses, with only about two-thirds reporting any blood pressure control measures and fewer than two-fifths reporting any blood glucose control measures, underscoring the need to strengthen continuity of care and integrated chronic disease management, including structured follow-up, patient counselling, and accessible health education. Such education and risk communication should be tailored to populations with lower educational attainment and limited health literacy to improve feasibility and effectiveness in real-world settings.

## Additional material


Online Supplementary Document
