# Erratum: Zou JT, Tao JL, Jiang J, Xiong CF, He B, Sun DW, Liu Y, Zhang DX. Hypertension-diabetes comorbidity in tropical Chinese adults. J Glob Health. 2026;16:04091.

**DOI:** 10.7189/jogh.16.1604091err1

**Published:** 2026-03-20

**Authors:** Journal of Global Health Editorial Team



Following the publication of the manuscript ‘Hypertension-diabetes comorbidity in tropical Chinese adults’ on 6 March 2026, two errors were identified in Figure 2. The positions of Panel A and Panel B were inadvertently reversed. In addition, several textual errors were present in both panels. 

In the previous version, Panel A (erroneously labelled as Panel B) consisted of the following values: Increased physical activity; Any blood pressure control measure; Any blood; Take medication as prescribed; Blood glucose; Blood blood; Take medication only when symptomatic. Panel B (erroneously labelled as Panel A) consisted of the following values: Insulin injection; Any blood; Increased physical activity; Blood glucose; Diet control; Oral medication. 

This has been corrected to: Panel A. Increased physical activity; Took any measures to control blood pressure; Salt reduction; Diet control; Take medication only when symptomatic; Blood pressure monitoring; Take medication as prescribed. Panel B. Insulin injection; Took any measures to control blood glucose; Increased physical activity; Blood glucose monitoring; Diet control; Oral medication.

The authors regret this error, take full responsibility, and apologise for any inconvenience it may have caused to the readers or the authors.

These corrections have been made to the online and PDF version of the manuscript on 20 March 2026, following the authors’ notice on 11 March 2026.

