# Supplementary material for: Hypertension-diabetes comorbidity in tropical Chinese adults
Source: J Glob Health. 2026 Mar 6;16:04091. doi: 10.7189/jogh.16.04091 (PMC12964091; doi:10.7189/jogh.16.04091)
Supplement: Online Supplementary Document [file jogh-16-04091-s001.pdf]

Supplement to: Zou JT, Tao JL, Jiang J, Xiong CF, He B, Sun DW, Liu Y, Zhang DX. Hypertension-diabetes comorbidity in tropical Chinese adults. J Glob Health. 2026;16:04091.

Figure S1. Factors associated with adopting any blood glucose control measure among participants with self-reported diabetes

Subgroup	aOR (95% CI)	P value
**Sex**		
Male	Reference	
Female	1.06 (0.73-1.67)	0.617
**Age group (years)**		
18-39	Reference	
40-59	0.81 (0.28-1.16)	0.222
>=60	0.87 (0.50-1.37)	0.505
**Ethnicity**		
Han	Reference	
Li	0.19 (0.07-0.73)	0.02
Other	0.83 (0.50-1.50)	0.5
**Urban / rural**		
Town	Reference	
Countryside	0.79 (0.53-1.78)	0.534
**Education status**		
Illiterate	Reference	
Primary school	1.07 (0.76-1.78)	0.792
Junior high school	1.46 (0.38-4.15)	0.18
High school	1.21 (0.53-2.29)	0.539
University or above	1.02 (0.95-2.03)	0.96
**Marital status**		
Unmarried	Reference	
Married/Cohabitation	1.54 (0.86-1.55)	0.406
Divorce/Widowhood	0.93 (0.87-3.03)	0.89
**BMI (kg/m2)**		
Normal	Reference	
Underweight	0.59 (0.27-1.42)	0.216
Overweight	1.02 (0.79-1.28)	0.807
Obese	1.04 (0.79-1.52)	0.82
**blood lipids**		
Normal	Reference	
Abnormal	1.18 (0.42-1.38)	0.194
**Smoking**		
Never smoked	Reference	
Current smoker	0.95 (0.77-1.45)	0.795
Former smoker	1.01 (0.92-1.53)	0.938
**Secondhand Smoke Exposure**		
none	Reference	
low frequency	0.83 (0.34-1.20)	0.301
high frequency	1.11 (0.65-1.85)	0.671
**Drinking**		
No drinking(past 12m)	Reference	
Former drinking(no 30d)	1.08 (0.57-1.48)	0.586
Drinking(past 30d)	0.66 (0.07-0.93)	0.022
**Physical activity intensity**		
No vigorous/moderate	Reference	
Vigorous only	1.42 (0.42-3.42)	0.397
Moderate only	2.13 (1.48-2.27)	0.043
vigorous + moderate	1.05 (0.88-2.39)	0.9

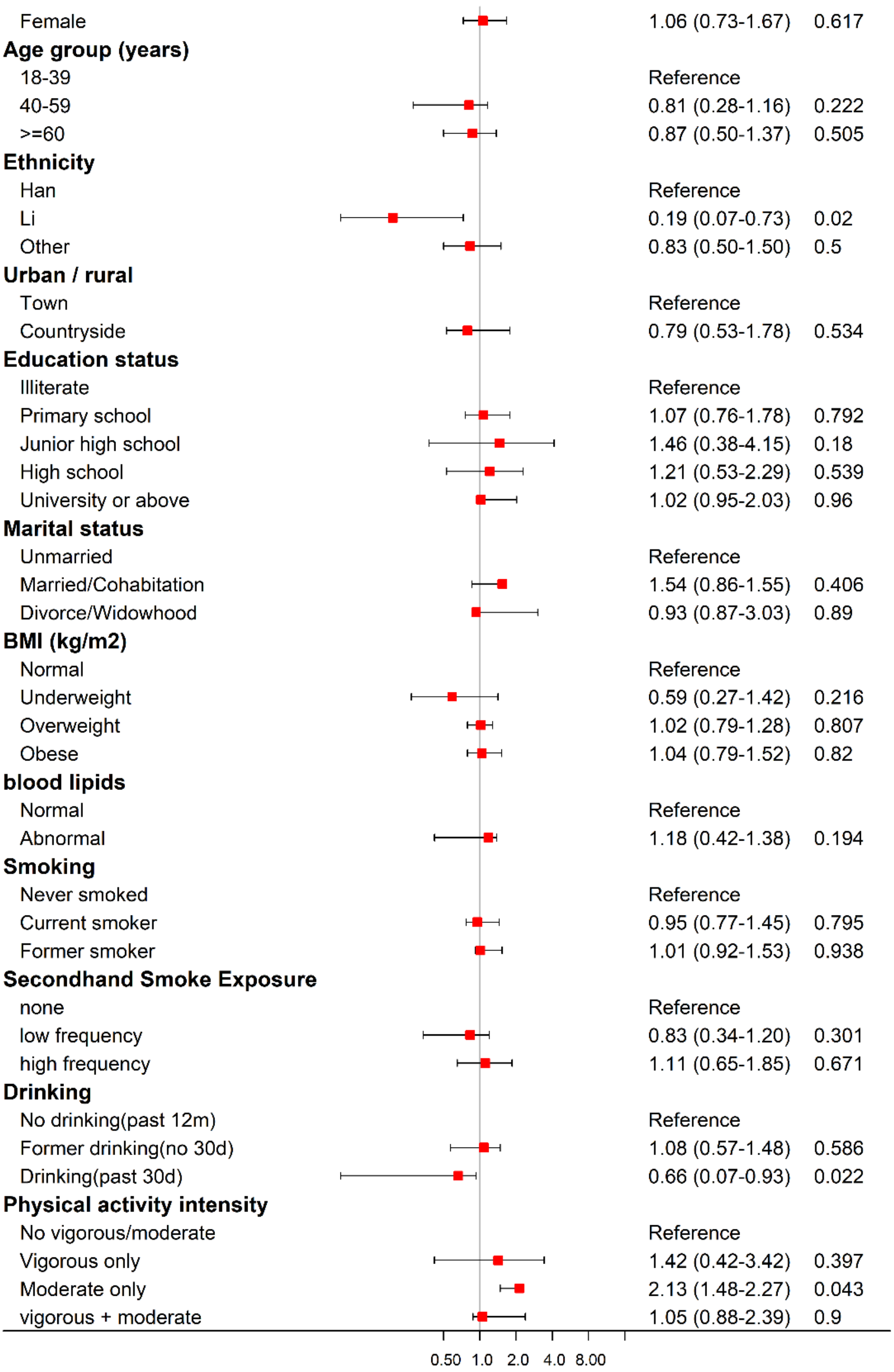

Table S1. Prespecified models (M1−M8): outcome definitions and stepwise covariate inclusion

**Variable**	**M1**	**M2**	**M3（Main model）**	**M4（HDC vs none）**	**M5（HDC vs only HTN/DM）**	**M6（only HTN vs none）**	**M7（only DM vs none）**	**M8（Objectively measured HDC）**
**Outcome definition**	**Hypertension–diabetes comorbidity (HDC) vs non-HDC.**	**Hypertension–diabetes comorbidity (HDC) vs non-HDC.**	**Hypertension–diabetes comorbidity (HDC) vs non-HDC.**	**HDC vs none (neither hypertension nor diabetes).**	**HDC vs single condition (hypertension only or diabetes only).**	**Hypertension only vs none.**	**Diabetes only vs none.**	**Objectively measured HDC vs non-HDC (self-reported physician diagnosis excluded).**
**X definition**	**Demographic covariates: gender, age group, ethnicity, urban/rural residence, education status, and marital status.**	**M1 + metabolic covariates: body mass index (BMI, kg/m²) and blood lipids (dyslipidemia).**	**M2 + lifestyle covariates: smoking status, second hand smoke exposure, alcohol drinking, and physical activity intensity.**	**Same covariates as M3 (12 covariates in total).**	**Same covariates as M3 (12 covariates in total).**	**Same covariates as M3 (12 covariates in total).**	**Same covariates as M3 (12 covariates in total).**	**Same covariates as M3 (12 covariates in total).**
**Unweighted valid N**	N=32857	N=32809	N =31350	N= 20160	N =14356	N =25674	N =19504	N =30293
**Unweighted invalid N（%）**	N (%) =0(0)	N (%) =48(0.1)	N (%) =1507(4.6)	N (%) =1001(4.7)	N (%) =646(4.3)	N(%) =1247 (4.6)	N (%) =981 (4.8)	N (%) =2564(7.8)

Notes: 1. Definition of hypertension: systolic blood pressure (SBP) ≥140 mmHg and/or diastolic blood pressure (DBP) ≥90 mmHg, or self-reported physician diagnosis.
2. Definition of diabetes: fasting plasma glucose (FPG) ≥7.0 mmol/L and/or 2-hour postprandial plasma glucose (2hPG) ≥11.1 mmol/L, or self-reported physician diagnosis.

3. Definition of HDC: presence of both hypertension and diabetes as defined above.
4. M4–M7: Separate binary contrasts derived from the four-category outcome (HDC / hypertension only / diabetes only / none); participants not belonging to the contrasted groups were excluded from each respective model.
5. M8: HDC was defined using objective measurements only (SBP/DBP and FPG/2hPG), excluding self-reported physician diagnosis; covariates were identical to M3.
6. Abbreviations: HDC, hypertension–diabetes comorbidity; HTN, hypertension; DM, diabetes mellitus; SBP, systolic blood pressure; DBP, diastolic blood pressure; FPG, fasting plasma glucose; 2hPG, 2-hour postprandial plasma glucose; BMI, body mass index.

Table S2. Associations of sociodemographic characteristics with HDC: survey-weighted multivariable logistic regression (M1)

Variable	Category	aOR(95%CI)	P
**Sex(Ref: Male)**	Female	0.631 (0.542–0.735)	＜0.001
**Age group(Ref: 18-39)**	40-59	5.752 (4.678–7.073)	＜0.001
	≥60	16.362 (13.462–19.887)	＜0.001
**Ethnicity(Ref: Han)**	Li	1.552 (1.212–1.989)	0.002
	Other	1.728 (1.084–2.756)	0.025
**Urban / rural (Ref: Town)**	Countryside	0.845 (0.666–1.072)	0.148
**Education status (Ref: Illiterate)**	Primary school	0.936 (0.687–1.274)	0.647
	Junior high school	0.825 (0.636–1.072)	0.135
	High school	0.830 (0.604–1.139)	0.224
	University or above	0.888 (0.661–1.193)	0.398
**Marital status (Ref: Unmarried)**	Married/Cohabitation	0.790 (0.570–1.095)	0.141
	Divorce/Widowhood	0.998 (0.775–1.285)	0.986

Note: M1-Model 1, aOR-adjusted odds ratio, CI-confidence interval, Ref-Reference.

Table S3. Associations of sociodemographic and metabolic factors with HDC: survey-weighted multivariable logistic regression (M2)

Variable	Category	aOR(95%CI)	P
**Sex(Ref: Male)**	Female	0.628 (0.548–0.721)	＜0.001
**Age group(Ref: 18-39)**	40-59	5.486 (4.402–6.838)	＜0.001
	≥60	17.049 (14.087–20.633)	＜0.001
**Ethnicity(Ref: Han)**	Li	1.647 (1.264–2.145)	0.001
	Other	1.582 (0.939–2.666)	0.079
**Urban / rural (Ref: Town)**	Countryside	0.859 (0.694–1.063)	0.146
**Education status (Ref: Illiterate)**	Primary school	0.900 (0.685–1.183)	0.417
	Junior high school	0.766 (0.605–0.970)	0.030
	High school	0.735 (0.548–0.987)	0.042
	University or above	0.796 (0.592–1.071)	0.120
**Marital status (Ref: Unmarried)**	Married/Cohabitation	0.737 (0.526–1.033)	0.073

	Divorce/Widowhood	0.949 (0.730–1.233)	0.669
**BMI (kg/m2)(Ref: Normal)**	Underweight	0.665 (0.515–0.858)	0.004
	Overweight	1.833 (1.640–2.049)	＜0.001
	Obese	2.680 (2.257–3.181)	＜0.001
**blood lipids(Ref: Normal)**	Abnormal	1.736 (1.577–1.911)	＜0.001

Note: aOR﹣adjusted odds ratio, CI﹣confidence interval, M2﹣Model 2, Ref﹣Reference.

Table S4. Sensitivity analyses using alternative outcome specifications

**Variable**	**Category**	**M3（Main model）**		**M4（HDC vs none）**		**M5（HDC vs only HTN/DM）**		**M6（only HTN vs none）**		**M7（only DM vs none）**	
		**aOR(95%CI)**	**P**	**aOR(95%CI)**	**P**	**aOR(95%CI)**	**P**	**aOR(95%CI)**	**P**	**aOR(95%CI)**	**P**
**Sex(Ref: Male)**	Female	0.637（0.550–0.736)	**<0.001**	0.511 (0.429–0.608)	**<0.001**	0.817 (0.719–0.927)	**0.005**	0.534 (0.479–0.595)	**<0.001**	0.744 (0.670–0.827)	**<0.001**
**Age group(Ref: 18-39)**	40-59	5.418 (4.285–6.850)	**<0.001**	7.450 (5.764–9.629)	**<0.001**	2.819 (2.210–3.595)	**<0.001**	2.769 (2.543–3.015)	**<0.001**	2.534 (1.962–3.273)	**<0.001**
	≥60	16.373 (13.376–20.041)	**<0.001**	35.539 (26.983–46.808)	**<0.001**	5.461 (4.466–6.677)	**<0.001**	7.390 (6.148–8.883)	**<0.001**	5.566 (4.324–7.165)	**<0.001**
**Ethnicity(Ref: Han)**	Li	1.634 (0.963–2.770)	0.004	1.979 (1.427–2.744)	**0.001**	1.310 (1.027–1.670)	0.032	1.517 (1.205–1.910)	0.002	1.314 (0.850–2.031)	0.197
	Other	1.599 (1.195–2.140)	0.066	1.657 (1.065–2.579)	0.029	1.630 (0.907–2.930)	0.094	0.914 (0.689–1.212)	0.501	0.909 (0.427–1.932)	0.787
**Urban / rural (Ref: Town)**	Countryside	1.000 (0.697–1.061)	0.144	1.000 (0.659–1.101)	0.199	0.854 (0.712–1.025)	0.084	1.007 (0.787–1.289)	0.953	1.024 (0.766–1.370)	0.859

**Education status (Ref: Illiterate)**	Primary school	0.869 (0.714–1.057)	0.144	0.741 (0.585–0.937)	0.017	1.005 (0.815–1.239)	0.959	0.702 (0.581–0.847)	0.001	0.922 (0.814–1.044)	0.179
	Junior high school	0.748 (0.621–0.902)	0.006	0.611 (0.482–0.774)	0.001	0.889 (0.751–1.053)	0.115	0.615 (0.489–0.773)	0.001	0.962 (0.806–1.149)	0.646
	High school	0.714 (0.570–0.893)	0.007	0.557 (0.424–0.733)	0.001	0.896 (0.719–1.116)	0.296	0.536 (0.422–0.681)	**<0.001**	0.940 (0.798–1.107)	0.426
	University or above	0.782 (0.593–1.033)	0.078	0.601 (0.423–0.853)	0.008	1.044 (0.843–1.294)	0.665	0.521 (0.362–0.750)	0.002	0.832 (0.672–1.030)	0.085
**Marital status (Ref: Unmarried)**	Married/Cohabitation	0.732 (0.518–1.035)	0.073	0.732 (0.492–1.090)	0.113	0.713 (0.518–0.981)	0.039	1.082 (0.870–1.346)	0.448	1.330 (1.024–1.728)	0.035
	Divorce/Widowhood	0.915 (0.699–1.197)	0.483	1.083 (0.779–1.505)	0.607	0.776 (0.596–1.010)	0.058	1.601 (1.229–2.086)	0.002	1.488 (1.049–2.110)	0.029
**BMI (kg/m2) (Ref: Normal)**	Underweight	0.667 (0.516–0.863)	0.005	0.551 (0.423–0.719)	**<0.001**	0.854 (0.666–1.095)	0.191	0.568 (0.455–0.708)	**<0.001**	0.905 (0.710–1.154)	0.390
	Overweight	1.831 (1.631–2.056)	**<0.001**	2.335 (2.016–2.704)	**<0.001**	1.410 (1.253–1.587)	**<0.001**	1.826 (1.682–1.984)	**<0.001**	1.494 (1.309–1.704)	**<0.001**
	Obese	2.679 (2.278–3.150)	**<0.001**	4.123 (3.386–5.022)	**<0.001**	1.689 (1.425–2.001)	**<0.001**	2.913 (2.573–3.298)	**<0.001**	2.306 (1.575–3.376)	**<0.001**
**blood lipids (Ref: Normal)**	Abnormal	1.740 (1.581–1.916)	**<0.001**	2.071 (1.847–2.322)	**<0.001**	1.418 (1.291–1.557)	**<0.001**	1.522 (1.358–1.706)	**<0.001**	1.602 (1.471–1.744)	**<0.001**

**Smoking (Ref: Never smoked)**	Current smoker	0.901 (0.698–1.162)	0.389	0.805 (0.585–1.108)	0.165	0.961 (0.767–1.205)	0.711	0.830 (0.702–0.981)	0.032	1.018 (0.844–1.228)	**0.839**
	Former smoker	1.072 (0.870–1.321)	0.484	1.162 (0.877–1.539)	0.268	1.029 (0.869–1.220)	0.716	1.105 (0.883–1.383)	0.352	1.051 (0.747–1.479)	0.755
**Secondhand Smoke Exposure (Ref: none)**	low frequency	0.782 (0.655–0.932)	0.010	0.740 (0.593–0.924)	0.012	0.845 (0.721–0.990)	0.039	0.786 (0.655–0.943)	0.014	0.965 (0.781–1.192)	0.721
	high frequency	0.935 (0.818–1.069)	0.296	0.953 (0.818–1.111)	0.509	0.937 (0.818–1.072)	0.313	1.006 (0.885–1.143)	0.921	0.978 (0.871–1.098)	0.683
**Drinking (Ref: No drinking in the past 12 months)**	Former drinking (no drinking in the past 30 days)	1.043 (0.937–1.160)	0.411	1.030 (0.882–1.202)	0.688	1.045 (0.926–1.180)	0.443	1.117 (0.946–1.319)	0.172	0.795 (0.621–1.017)	0.065
	Drinking in the past 30 days	1.144 (1.004–1.304)	0.045	1.264 (1.063–1.502)	0.012	1.024 (0.913–1.148)	0.659	1.322 (1.161–1.505)	0.001	0.947 (0.749–1.197)	0.622
**Physical activity intensity (Ref: No vigorous- or moderate-intensity activity)**	Vigorous-intensity activity only	0.946 (0.744–1.202)	0.621	0.850 (0.634–1.139)	0.249	1.014 (0.795–1.294)	0.903	0.940 (0.677–1.306)	0.691	0.784 (0.522–1.176)	0.215
	Moderate-intensity activity only	0.947 (0.785–1.142)	0.538	0.951 (0.718–1.260)	0.704	0.942 (0.806–1.102)	0.423	1.025 (0.746–1.408)	0.870	0.945 (0.834–1.070)	0.338
	Both vigorous- and moderate-intensity activity	0.907 (0.798–1.030)	0.120	0.889 (0.739–1.070)	0.193	0.930 (0.801–1.080)	0.310	0.967 (0.774–1.208)	0.746	0.892 (0.784–1.015)	0.078

Note: M3–M7, models 3–7; aOR - adjusted odds ratio; CI - confidence interval; Ref - reference group.

Table S5. Sensitivity analysis using objectively measured HDC as the outcome

Variable	Category	M3（Main model）		M8（Objectively measured HDC）	
		aOR(95%CI)	P	aOR(95%CI)	P
**Gender(Ref: Male)**	Female	0.637（0.550－0.736)	**<0.001**	0.561 (0.480-0.655)	＜0.001
**Age group(Ref: 18-39)**	40-59	16.373 (13.376－20.041)	**<0.001**	5.173 (4.313-6.204)	＜0.001
	≥60	5.418 (4.285－6.850)	**<0.001**	12.730 (10.357-15.648)	＜0.001
**Ethnicity(Ref: Han)**	Li	1.634 (0.963－2.770)	0.004	1.763 (1.298-2.395)	0.002
	Other	1.599 (1.195－2.140)	0.066	1.715 (0.918-3.204)	0.085
**Urban and rural (Ref: Town)**	Countryside	1.000 (0.697－1.061)	0.144	0.936 (0.811-1.080)	0.335
**Education status (Ref: Illiterate)**	Primary school	0.869 (0.714－1.057)	0.144	0.799 (0.607-1.053)	0.102
	Junior high school	0.748 (0.621－0.902)	0.006	0.693 (0.509-0.944)	0.024
	High school	0.714 (0.570－0.893)	0.007	0.613 (0.437-0.858)	0.008
	University or above	0.782 (0.593－1.033)	0.078	0.667 (0.462-0.961)	0.033
**Marital status (Ref: Unmarried)**	Married/Cohabitation	0.732 (0.518－1.035)	0.073	0.671 (0.501-0.897)	0.011
	Divorce/Widowhood	0.915 (0.699－1.197)	0.483	0.846 (0.689-1.039)	0.102
**BMI (kg/m2) (Ref: Normal)**	Underweight	0.667 (0.516－0.863)	0.005	0.697 (0.573-0.848)	0.002
	Overweight	1.831 (1.631－2.056)	**<0.001**	1.849 (1.640-2.085)	＜0.001
	Obese	2.679 (2.278－3.150)	**<0.001**	2.644 (2.132-3.280)	＜0.001
**blood lipids (Ref: Normal)**	Abnormal	1.740 (1.581－1.916)	**<0.001**	1.981 (1.813-2.164)	＜0.001
**Smoking (Ref: Never smoked)**	Current smoker	0.901 (0.698－1.162)	0.389	0.893 (0.693-1.150)	0.347
	Former smoker	1.072 (0.870－1.321)	0.484	1.020 (0.802-1.297)	0.859
**Secondhand Smoke Exposure (Ref: none)**	low frequency	0.782 (0.655－0.932)	0.010	0.844 (0.708-1.005)	0.055
	high frequency	0.935 (0.818－1.069)	0.296	0.949 (0.815-1.105)	0.470
**Drinking (Ref: No drinking in the past 12 months)**	Former drinking (no drinking in the past 30 days)	1.043 (0.937－1.160)	0.411	1.030 (0.892-1.188)	0.665

	Drinking in the past 30 days	1.144 (1.004 – 1.304)	0.045	1.299 (1.109–1.522)	0.004
**Physical activity intensity (Ref: No vigorous- or moderate-intensity activity)**	Vigorous-intensity activity only	0.946 (0.744 – 1.202)	0.621	1.092 (0.832–1.433)	0.496
	Moderate-intensity activity only	0.947 (0.785 – 1.142)	0.538	1.047 (0.828–1.324)	0.678
	Both vigorous- and moderate-intensity activity	0.907 (0.798 – 1.030)	0.120	1.008 (0.857–1.186)	0.915

Note: aOR–adjusted odds ratio, CI–confidence interval, HDC–hypertension–diabetes comorbidity, M3–Model 3, M8–Model 8, Ref–reference.

Table S6. Factors associated with adopting control measures among participants with self-reported physician-diagnosed hypertension

Group	Variable	Category	Reference	Unweighted n	aOR (Exp(B))	95% CI	P value
**Demographics**							
	Sex	Male	**Yes**	2293	—	—	—
	Sex	Female		2764	0.84	(0.68-1.03)	0.089
	Age group	18–39	**Yes**	247	—	—	—
	Age group	40–59		1747	2.893	(2.23-3.75)	< 0.001
	Age group	≥60		3063	6.318	(5.08-7.86)	< 0.001
	Ethnicity	Han	**Yes**	3979	—	—	—
	Ethnicity	Li		1006	3.072	(2.11-4.48)	< 0.001
	Ethnicity	Other		72	1.155	(0.55-2.44)	0.682
	Urban/Rural	Urban/Town	**Yes**	1275	—	—	—
	Urban/Rural	Rural/Countryside		3782	0.548	(0.34-0.89)	0.019
	Education status	Illiterate	**Yes**	876	—	—	—
	Education status	Primary school		1471	1.122	(0.73-1.73)	0.570

	Education status	Junior high		1616	0.941	(0.61-1.46)	0.769
	Education status	High school/secondary		861	0.999	(0.58-1.72)	0.997
	Education status	College+		233	0.711	(0.36-1.40)	0.294
	Marital status	Single	**Yes**	158	—	—	—
	Marital status	Married/cohabiting		4560	1.015	(0.58-1.78)	0.955
	Marital status	Divorced/widowed		339	0.979	(0.65-1.48)	0.912
**Metabolic**							
	BMI (WHO category)	Normal (18.5–24.9)	**Yes**	2717	—	—	—
	BMI (WHO category)	Underweight (<18.5)		184	0.687	(0.50-0.95)	0.027
	BMI (WHO category)	Overweight (25.0–29.9)		1818	1.479	(1.24-1.76)	< 0.001
	BMI (WHO category)	Obese (≥30.0)		337	1.640	(1.28-2.10)	0.001
	Blood lipids	Normal lipids	**Yes**	2561	—	—	—
	Blood lipids	Dyslipidemia		2490	1.067	(0.88-1.30)	0.481
**Lifestyle**							
	Smoking status	Never	**Yes**	3653	—	—	—
	Smoking status	Current		1011	0.753	(0.44-1.30)	0.283
	Smoking status	Former		393	1.206	(0.88-1.65)	0.220
	Secondhand smoke exposure	None	**Yes**	2181	—	—	—
	Secondhand smoke exposure	Low (1–3 days/week)		556	1.329	(0.85-2.08)	0.193
	Secondhand smoke exposure	High (≥4 days/week or daily)		2141	0.960	(0.72-1.28)	0.763
	Drinking status	No drinking in past 12 months	**Yes**	3345	—	—	—

	Drinking status	Former drinker		425	0.887	(0.66-1.19)	0.389
	Drinking status	Drank in past 30 days		1287	0.747	(0.56-1.00)	0.051
	Physical activity intensity	None	**Yes**	1750	—	—	—
	Physical activity intensity	Vigorous only		158	1.151	(0.61-2.16)	0.634
	Physical activity intensity	Moderate only		1801	1.675	(0.79-3.56)	0.162
	Physical activity intensity	Both vigorous & moderate		1348	1.040	(0.56-1.92)	0.891

Note:1.This cross-sectional analysis examines factors associated with adopting control measures among diagnosed participants; estimates describe associations and do not imply causality (reverse causation is possible).

2.Sample: Participants with self-reported physician-diagnosed hypertension(n=5057).

3.Outcome:took any measures to control blood pressure.

4. aOR – adjusted odds ratio, CI – confidence interval.

Table S7. Factors associated with adopting control measures among participants with self-reported physician-diagnosed diabetes

Group	Variable	Category	Reference	Unweighted n	aOR (Exp(B))	95% CI	P value
**Demographics**							
	Sex	Male	**Yes**	1102	—	—	—
	Sex	Female		1263	1.061	(0.73-1.67)	0.617
	Age group	18–39	**Yes**	97	—	—	—
	Age group	40–59		797	0.807	(0.28-1.16)	0.222
	Age group	≥60		1471	0.866	(0.50-1.37)	0.505
	Ethnicity	Han	**Yes**	2009	—	—	—

	Ethnicity	Li		317	0.190	(0.07-0.73)	0.020
	Ethnicity	Other		39	0.828	(0.50-1.50)	0.500
	Urban/Rural	Urban/Town	**Yes**	783	—	—	—
	Urban/Rural	Rural/Countryside		1582	0.786	(0.53-1.78)	0.534
	Education status	Illiterate	**Yes**	366	—	—	—
	Education status	Primary school		601	1.066	(0.76-1.78)	0.792
	Education status	Junior high		749	1.459	(0.38-4.15)	0.180
	Education status	High school/secondary		491	1.205	(0.53-2.29)	0.539
	Education status	College+		158	1.017	(0.95-2.03)	0.960
	Marital status	Single	**Yes**	54	—	—	—
	Marital status	Married/cohabiting		2174	1.540	(0.52-4.60)	0.406
	Marital status	Divorced/widowed		137	0.926	(0.87-3.03)	0.890
**Metabolic**							
	BMI (WHO category)	Normal (18.5–24.9)	**Yes**	1372	—	—	—
	BMI (WHO category)	Underweight (<18.5)		86	0.591	(0.27-1.42)	0.216
	BMI (WHO category)	Overweight (25.0–29.9)		770	1.024	(0.79-1.28)	0.807
	BMI (WHO category)	Obese (≥30.0)		137	1.041	(0.79-1.52)	0.820
	Blood lipids	Normal lipids	**Yes**	1119	—	—	—

	Blood lipids	Dyslipidemia		1237	1.175	(0.42-1.38)	0.194
**Lifestyle**							
	Smoking status	Never	**Yes**	1692	—	—	—
	Smoking status	Current		504	0.950	(0.77-1.45)	0.795
	Smoking status	Former		169	1.015	(0.92-1.53)	0.938
	Secondhand smoke exposure	None	**Yes**	1059	—	—	—
	Secondhand smoke exposure	Low (1–3 days/week)		272	0.834	(0.34-1.20)	0.301
	Secondhand smoke exposure	High (≥4 days/week or daily)		925	1.108	(0.65-1.85)	0.671
	Drinking status	No drinking in past 12 months	**Yes**	1709	—	—	—
	Drinking status	Former drinker		189	1.083	(0.57-1.48)	0.586
	Drinking status	Drank in past 30 days		467	0.664	(0.07-0.93)	0.022
	Physical activity intensity	None	**Yes**	845	—	—	—
	Physical activity intensity	Vigorous only		63	1.424	(0.42-3.42)	0.397
	Physical activity intensity	Moderate only		971	2.127	(1.48-2.27)	0.043
	Physical activity intensity	Both vigorous & moderate		486	1.050	(0.88-2.39)	0.900

Note:1.This cross-sectional analysis examines factors associated with adopting control measures among diagnosed participants; estimates describe associations and do not imply causality (reverse causation is possible).

2.Participants with self-reported physician-diagnosed diabetes(n=2365)

3.Outcome:took any measures to control blood glucose

4. aOR – adjusted odds ratio, CI – confidence interval.
